# Superhydrophilic 2D Carbon Nitrides Prepared by Direct Chemical Vapor Deposition

**DOI:** 10.1002/smsc.202200099

**Published:** 2023-03-09

**Authors:** Quoc Huy Thi, Ping Man, Lingli Huang, Xin Chen, Jiong Zhao, Thuc Hue Ly

**Affiliations:** ^1^ Department of Chemistry and Center of Super-Diamond and Advanced Films (COSDAF) City University of Hong Kong Kowloon Hong Kong China; ^2^ City University of Hong Kong Shenzhen Research Institute Shenzhen 518000 China; ^3^ Department of Applied Physics The Hong Kong Polytechnic University Kowloon Hong Kong China; ^4^ Shenzhen Research Institute The Hong Kong Polytechnic University Shenzhen 518000 China

**Keywords:** carbon nitrides, chemical vapor deposition, super hydrophilicity

## Abstract

Surface wetting greatly impacts the performances of many photocatalysts in a water/humid‐involved medium. Carbon nitrides and its isotopes, as emerging metal‐free low‐cost photocatalysts for water splitting, usually require strong chemical or irradiation treatments to obtain highly hydrophilic surfaces, which can undermine their photocatalytic performances. Herein, an alternative method for the direct synthesis of superhydrophilic carbon nitride thin films (CN_*x*_, *x* ≈ 0.86–1.04) and graphitic carbon nitride powder (g‐C_3_N_4_) by using chemical vapor deposition is proposed. Less than 5° contact angle with water is accessible on both the surface of the as‐grown CN_*x*_ thin films and the membranes made from the g‐C_3_N_4_ powder. It is found that the remarkable wetting property can be attributed to the spontaneous hydrophilic functionalization group (e.g., –OH, –NO_*x*_, = O) supplied by a constant multielemental air flow. The abundant CN triple bonds also promote needle‐shaped nanostructures on the 2D surfaces, which enhances their chemical wettability. Finally, the tremendous potential of this novel technique for direct synthesis of superhydrophilic carbon nitride in photocatalysis applications is demonstrated.

## Graphical abstract

A new method for synthesizing superhydrophilic carbon nitride (CN_*x*_) thin films and graphitic carbon nitride powder (g‐C_3_N_4_) via chemical vapor deposition is proposed. The superior wetting behavior is attributed to the hydrophilic functionalization groups and the abundant C≡N triple bonds that promote needle‐shaped nanostructures on the 2D surfaces. The promising potential of this novel technique in photocatalysis applications is demonstrated.

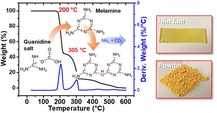

## 1 Introduction

Carbon nitride (CN_*x*_) materials are well known as emerging metal‐free photocatalysts for water splitting,^[^ 1, 2, 3 ^]^ energy storage,^[^ 4, 5 ^]^ and water filtration membranes.^[^ 6, 7 ^]^ Their prolific allotropes^[^ 3, 8 ^]^ with rich surface properties^[^ ^9^ ^]^ offer a high flexibility in structural and property modification,^[^ ^10^ ^]^ e.g., bandgap modulations by surface functionalization^[^ ^11^ ^]^ or carbon/nitrogen (C/N) ratio.^[^ ^12^ ^]^ In contrast, the rich allotropes make it difficult to directly modify the properties during synthesis process, as a little change may lead to varied and unpredictable products. Meanwhile, the functionalization could be realized by second‐time growth^[^ ^11^ ^]^ or posttreatment^[^ ^13^ ^]^ to convert the common structure like graphitic carbon nitrides (g‐C_3_N_4_) to the desired structures, however, with long processing time, high production loss, and inevitable hazardous chemical usage.

Superhydrophilicity benefits many applications working in the water/humid‐involved environments,^[^ ^14^ ^]^ from the self‐cleaning outdoor window surfaces,^[^ ^15^ ^]^ to the high performance photocatalysts and membranes in various studies.^[^ 16, 17, 18 ^]^ The contribution of superhydrophilicity to the surface applications can be categorized into two factors: First, by increasing the attraction to water molecules which directly speed up the water splitting reactions^[^ 19, 20 ^]^ or water transportation through the membrane;^[^ 17, 21 ^]^ Second, by self‐cleaning effect preventing the contaminations of catalytic surface or/and fouling effect on the membrane applications.^[^ 16, 18 ^]^ Therefore, increasing hydrophilicity without interfering their intrinsic properties of target materials is a major concern of many past studies.

Unfortunately, most of the carbon‐based materials such as graphene and its analogous are hydrophobic owing to their large inert surfaces.^[^ 22, 23 ^]^ Doping nitrogen to carbon‐based structure or implanting oxygen‐based functional groups on surfaces can increase the hydrogen bonding between surface and water molecules. Note that the CN_*x*_ materials are alternative metal‐free photocatalysts with narrow bandgap.^[^ ^1^ ^]^ Previous literature has reported that contact angle (CA) between water and the ideal condensed g‐C_3_N_4_ was 53.5°,^[^ ^24^ ^]^ meanwhile the 2D CN_*x*_ thin films prepared by bottom‐up growth had water wettability of 60°–80°.^[^ ^25^ ^]^ The CN_*x*_ film could be converted to hydrophobic by increasing surface porosity, resulting from the modification of precursor ratio or the source‐substrate distances.^[^ 12, 24, 25 ^]^ However, the superhydrophilic 2D CN_*x*_ or g‐C_3_N_4_ membranes have not been acquired yet by direct synthesis. Alternatively, the post‐synthesis functionalized CN_*x*_ surfaces with oxygenated molecules did improve the hydrophilicity, but their CA with water was over 24°.^[^ ^26^ ^]^ Moreover, embedding functional groups after synthesis caused unwanted disruptions to the initial lattice structure that significantly reduced their durability.

The carbon nitrides can be simply obtained by doping nitrogen into graphite. Besides, thermal polymerization of melamine (C_3_N_3_(NH_2_)_3_) or other N‐rich compounds like urea (CN_2_OH_4_), cyanamide (CN_2_H_2_) and its dimer (dicyandiamide, C_2_N_4_H_4_) can produce the powder of g‐C_3_N_4_.^[^ 1, 27, 28 ^]^ However, these methods cannot control the surface wettability of products directly but required some complicated posttreatments to functionalize the origin surface.^[^ ^26^ ^]^ Therefore, a new synthesis method that can spontaneously yield superhydrophilic surface is essential and pressing. In this work, we developed a new method for direct synthesis of the superhydrophilic CN_*x*_ thin films on hydrophobic substrate and highly crystalline superhydrophilic powder of g‐C_3_N_4_ using the chemical vapor deposition (CVD). The CA with water of the CN_*x*_ thin films and the membranes made from g‐C_3_N_4_ powder reaches below 5°, which is inaccessible via direct growth before. In addition, different surface textures and water wettability could be tuned by controlling the thermal condensation temperature of 450–600 °C. The new superhydrophilic CN_*x*_ structure has shown their potential as metal‐free photocatalyst for hydrogen evolution reaction (HER).

## 2 Results and Discussions

**Figure 1:**
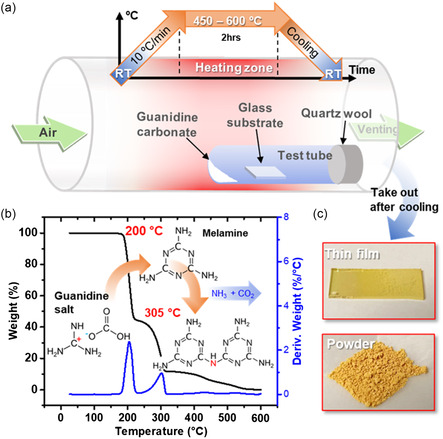
Synthesis of carbon nitrides (CN_*x*_). a) Schematic illustrates the experimental setup and the heating process for carbon nitrides synthesis. b) Thermogravimetric analysis (TGA) for decomposition of guanidine carbonate as precursor and the polycondensation of carbon nitrides during heating process. c) Digital pictures of the final products of CN_*x*_ thin film and powder taken out from CVD synthesis chamber after cooling.

Our experimental setup was schematically illustrated in **Figure** **1**a. Guanidine carbonate salt (NH_2_C(=NH)NH_2_·½H_2_CO_3_) as precursor was placed in the end of a quartz test tube. An amorphous glass substrate was positioned separately at a certain distance from the precursor. The substrate position was optimized to obtain the transparent uniform yellow thin film after synthesis (Figure S1, Supporting Information). The test tube was sealed with quartz wool and put into a tube furnace. During synthesis, the tube furnace was heated up by 10 °C min^−1^ to desired temperature and annealed for 2 h and then naturally cooled down to room temperature. Before initiating the heating process, an air flow of 100 mL min^−1^ was introduced for 30 min to eliminate the humidity in the tube furnace. This air flow was maintained constant until the synthesis was completed.

Chemical reactions in the synthesis process were characterized by thermogravimetric analysis (TGA) in Figure 1b. The derivations of weight loss vs temperature showed two distinguished peaks at 200 and 305 °C. The first peak at 200 °C indicated the decomposition of guanidine carbonate into guanidium (C(CH_2_)_3_^+^) and carbonate (H_2_CO_3_) molecules. Additionally, (C(CH_2_)_3_^+^) ions were linked together to form melamine and released ammonia (NH_3_).^[^ ^29^ ^]^ When the temperature ramped above 200 °C, the derivative curve fell to zero and gradually increased thereafter, indicating that the decomposition was completed. This was followed by an acceleration in the polymerization of guanidine ions to form volatile melamine as the temperature increased. The rise of derivative curve was also relevant with the increase of weight loss due to sublimation of melamine as well as NH_3_ release. The derivation of weight loss to temperature reached the second peak at 305 °C, corresponding to the maximum melamine sublimation rate.

The temperature was ramped up to 450, 500, 550, and 600 °C and held for two hours in separated synthesis experiments, before naturally cooling to room temperature (see Experimental Section). Since the precursor decomposition was complete at 305 °C, further annealing at higher temperatures was primarily employed to sublime melamine onto the downstream glass substrate and to promote the polycondensation of melamine ions into larger melem ions, which serve as the primary units of polyheptazine (g‐C_3_N_4_). Finally, the transparent yellow thin film deposited on glass substrate and the yellow solid powder at the end of test tube were collected after annealing for further characterization (Figure 1c).

**Figure 2:**
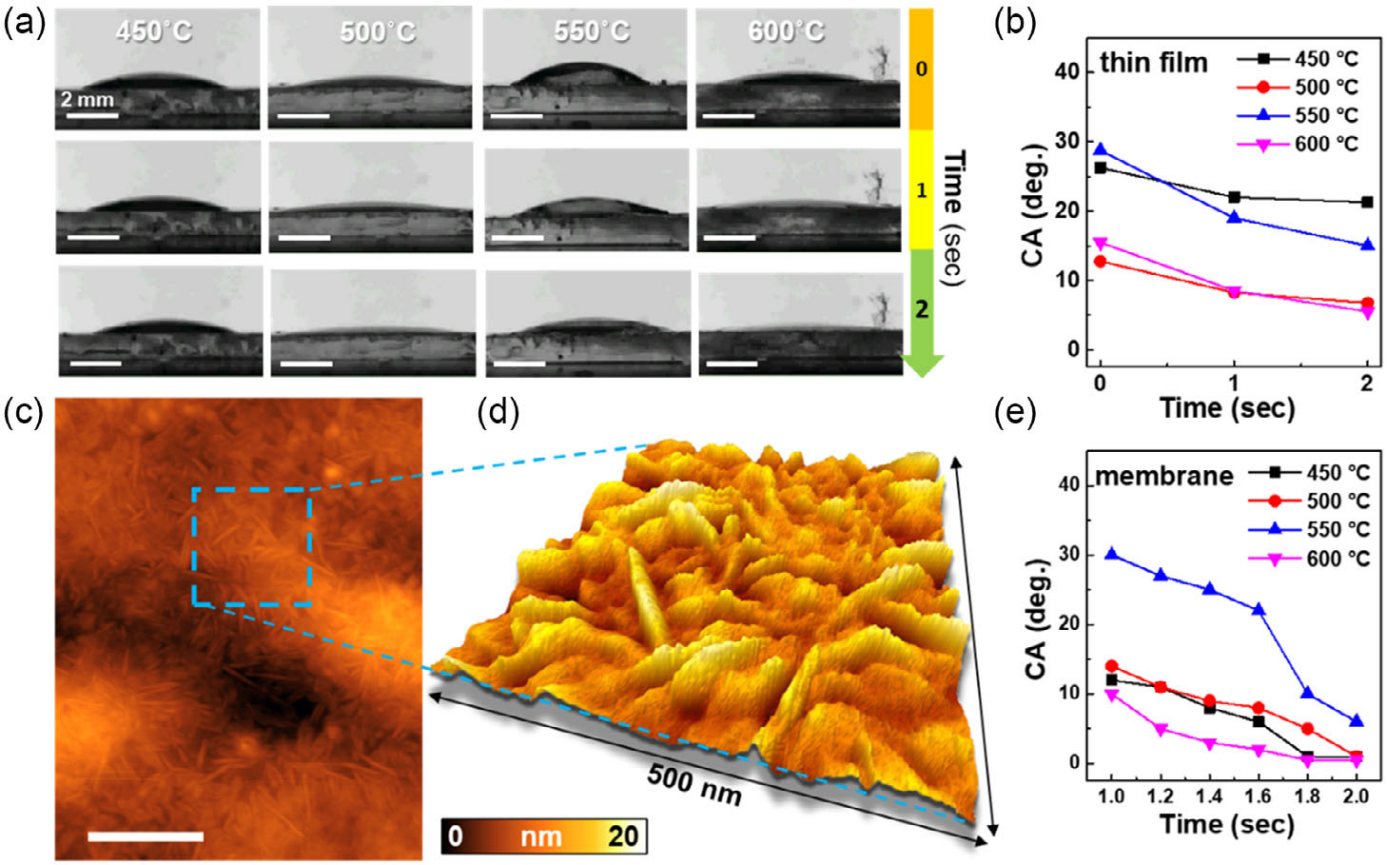
Superhydrophilicity of CN_*x*_ thin films and membranes. a) Series of snapshots showing the evolution of contact angle (CA) between the surface of the CN_*x*_ thin films and the water droplet over time. Scale bar = 2 mm. b) The time‐dependent CA of a water droplet on the surface of the CN_*x*_ thin films. c) AFM topographic image of CN_*x*_ thin films deposited at 500 °C, which contained needle‐shaped texture on 2D surface. Scale bar = 500 nm. d) The 3D layout of zoom‐in area corresponding to the blue square dashed line in (c). e) The time‐dependent CA of water droplet on surface of membranes made from CN_*x*_ powder.

The wettability of CN_*x*_ thin films and membranes made from powder samples (see Experimental Section and Figure S2, Supporting Information) were unravelled by the CA measurement with water droplet (**Figure** **2**). Surprisingly, both CN_*x*_ thin film and the membranes exhibited superhydrophilic behaviors. From the CA of water droplets on the surface of CN_*x*_ thin films (Figure 2a,b), two groups with distinct wetting behaviors were identified. The first group comprises samples synthesized at 450 and 550 °C, while the second group consists of samples synthesized at 500 and 600 °C. Upon initial contact, the first group exhibited a water CA of ≈25°–30°, while the second group had a low CA of 10°–15°. Two seconds after initial contact, the water droplets on the 450 and 550 °C films maintained a CA of ≈15°–20°, while the 500 and 600 °C films showed a CA with water below 10°. The different contrasts on thin film sample under optical microscope (OM) (Figure S3, Supporting Information) were also correlated with their wetting property. Relation between CA value and surface morphology can be described by Wenzel's equation^[^ ^30^ ^]^ as shown later

(1) $$\text{cos}\theta_{m}=R\text{cos}\theta_{y}$$

where *R* is the ratio between the actual solid surface area and the nominal surface area; *θ*_y_ and *θ*_m_ are the Young contact angle^[^ ^31^ ^]^ and measured CA of actual solid surface with water, respectively. According to Wenzel's Equation (1), an increase in surface roughness leads to an increase in chemical wettability. The difference in CA between the 600 and 500 °C films of 1°–2° is negligible, suggesting that their surfaces had similar morphology and chemistry. Additionally, the initial CA of the 550 °C film (28.75°) was higher than that of the 450 °C film (26.25°). After 2 s, the droplets on the surface stabilized, and the final CA of the 550 °C film (15°) was significantly lower than the final CA of the 450 °C film (21.25°). This phenomenon can be explained by the relation between CA and surface porosity (*f*_p_) described by Cassie–Baxter's equation (2)^[^ ^32^ ^]^

(2) $$f_{p}=1-\left[\left(\text{cos}\theta_{m}+1\right)/\left(\text{cos}\theta_{y}+1\right)\right]$$

The final variation in CA between the 450 and 550 °C films was attributed to the fact that the 450 °C film had a higher porosity than the 550 °C film, as evidenced by the cross‐sectional scanning electron microscope (SEM) images (Figure S4, Supporting Information). The cross‐sectional SEM image of the 450 °C film revealed a high level of structural porosity, and the porosity decreased as the thickness decreased by increasing the polycondensation temperature while using the same annealing time of 2 h, which was consistent with the final CA measurement value (Figure 2b). Next, the surfaces of CN_*x*_ thin films were characterized by AFM (Figure 2c,d and S5, Supporting Information), and the surface roughness and CA value of each thin film sample are summarized in Table S1, Supporting Information. Specially, the surface of CN_*x*_ thin films synthesized at 500 °C were composed of 1D needle‐shaped structures. (Figure 2c,d). The 1D structure on the 2D surface greatly increased the surface roughness resulting in the enhancement of hydrophilicity according to the Wenzel's Equation (1), which lined up with the smallest CA with water of the 500 °C thin films. Besides, due to the high thermal expansion of glass substrate at 600 °C, the CN_*x*_ thin films suffered heavy delamination from underlying substrate (Figure S4d, Supporting Information), preventing the accurate observation of surface and thickness. Moreover, the membranes exhibited the lowest CA with water of 0.5°–1° (Figure 2e). Although the trend of water CA with surfaces largely agreed with theories, the experimental results exceeded the calculated values. These results implied that the annealing temperature modulated the surface morphology and surface chemistry, which could collectively influence the hydrophilicity of both the CN_*x*_ thin films and the powder samples.

**Figure 3:**
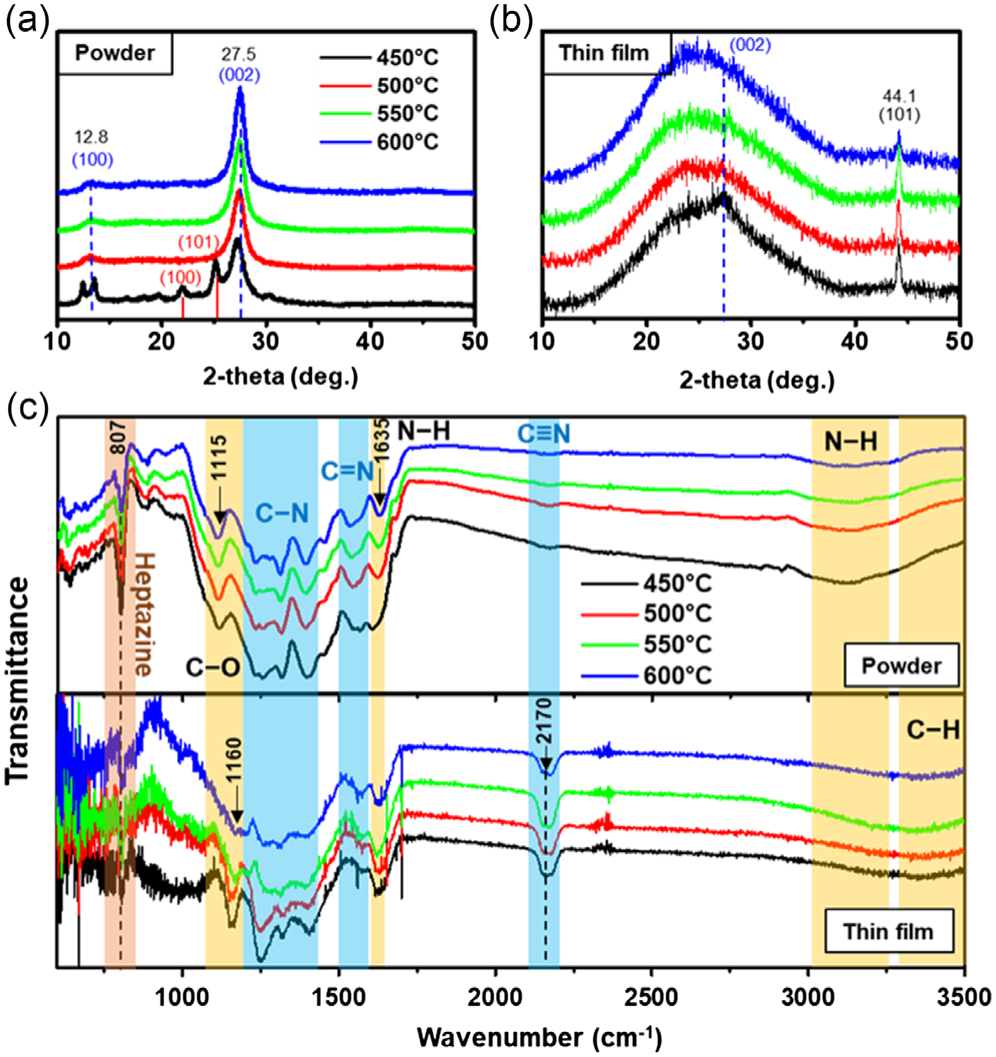
Crystalline structure and chemical bonding of carbon nitrides powder and thin films. a,b) X‐ray diffraction (XRD) spectra of powder (a) and thin films (b) deposited on glass substrates at different annealing temperature. The red lines marked for the peaks of triazine structure and blue dashed line marked for the peaks of heptazine structure. c) Fourier‐transform infrared (FT‐IR) spectra of powder and thin films deposited on glass substrates at different annealing temperature.

To further understand the physical structure and chemical bonding in the CN_*x*_ thin films and membranes, we employed the X‐ray diffraction (XRD) and Fourier‐transform infrared (FT‐IR) measurements on both powder and thin film samples. The yellow powder samples were identified as polyheptazine g‐C_3_N_4_ (Figure S6a, Supporting Information), referring to the diffraction peaks at 2*θ* = 12.8° and 27.5° (**Figure** **3**a), which corresponded to (100) and (002) planes of the polyheptazine layered structure,^[^ 1, 33 ^]^ respectively. The (100) peak corresponded to in‐plane distance of 0.68 nm between the neighboring heptazine units, and the (002) peak indicated the spacing of 0.32 nm between the graphitic layers.^[^ ^1^ ^]^ In particular, the 450 °C powder exhibited additional peaks at 2*θ* = 22° and 25.2°, which were attributed to the diffraction from (100) and (101) planes of triazine based g‐C_3_N_4_ (Figure S6b, Supporting Information).^[^ ^33^ ^]^ XRD analysis revealed that the g‐C_3_N_4_ powder synthesized at 450 °C consisted of both triazine and heptazine phase, while the powder synthesized at higher temperature only contained the heptazine phase. This is because the heptazine networks are more stable than the triazine networks,^[^ ^34^ ^]^ and thus the products formed heptazine networks at higher annealing temperatures. In contrast, the CN_*x*_ films exhibited lower crystallinity in which the (002) peak of graphitic structure was barely observed under the broad diffraction peak from the amorphous glass. An additional peak at 44.1° was observed, which corresponded to the diffraction from (101) planes^[^ ^35^ ^]^ of graphite layered structure (Figure 3b).

In Figure 3c, the FT‐IR result of powder and thin films samples had a distinct peak at 807 cm^−1^ referring to the vibration of heptazine groups,^[^ ^36^ ^]^ which agreed with the XRD result. The CN hexagonal rings were represented by high‐intensity multipeak in the range of 1200–1400 cm^−1^, which was correlated to the stretching of C−N and C=N bonds.^[^ ^1^ ^]^ The peak at 1115 cm^−1^ was correlated to C−O stretching which evidenced to the presence of oxygen‐based functional groups. In addition, the peak in 1635 cm^−1^ was correlated with N−H bending, and the broad peak range of 3000–3300 cm^−1^ was related to N−H stretching.^[^ 3, 24 ^]^ From the FT‐IR result can be seen that our direct growth samples contained sufficient O‐based groups and NH groups, which mainly contributed to their superhydrophilic behavior. Notably, the coexistence of O‐based groups and NH groups was rare because oxygen would react selectively with hydrogen rather than binding to the surface. This limited the proportion of O‐based groups embedded to the surface. However, this limitation was absent in our synthesis because these functional groups were spontaneously implanted during the polycondensation of samples, rather than being embedded after synthesis. The chemical bonds in thin films were similar to these in powder samples except for the higher proportion of C≡N bonds peaking at 2170 cm^−1^, which responded to the 1D texture on CN_*x*_ thin films (Figure 2c,d). Moreover, the large amount of functional groups implanted also caused corrugation to the surface itself.^[^ ^37^ ^]^

**Figure 4:**
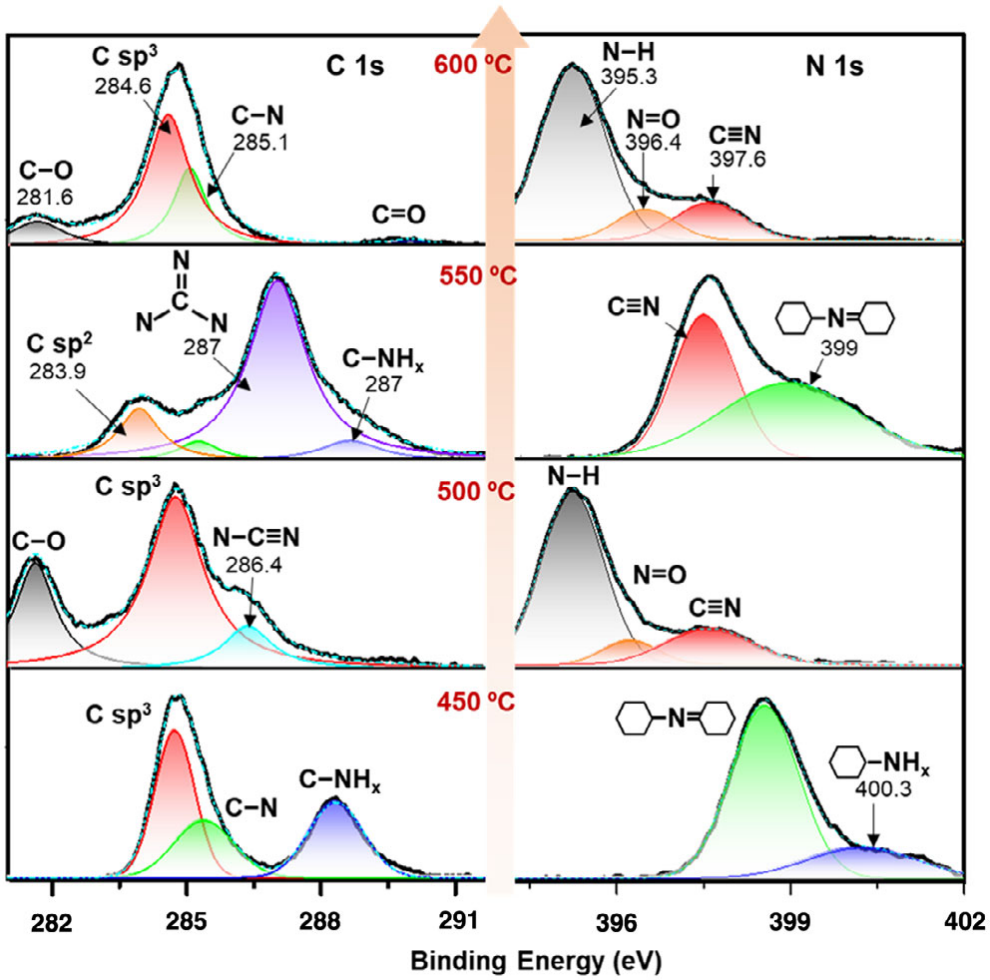
X‐ray photoelectron spectroscopy (XPS) characterizations of CN_*x*_ thin films deposited on glass substrate at different annealing temperature. The XPS spectrum peak fitting of carbon 1s core‐level (left) and nitrogen 1s core‐level (right) of CN_*x*_ thin films deposited on glass substrate at 450, 500, 550, and 600 °C.

**Figure** **4** displayed the X‐ray photoelectron spectroscopy (XPS) characterization for the chemical bonds on surface of CN_*x*_ thin films. The highest peak of C 1s core level in 600, 500, and 450 °C thin films was centered at 284.7 eV with the half‐maximum full width of 1.5 eV, corresponding to the tetrahedral C−C sp^3^ bonding.^[^ ^14^ ^]^ In contrast, the 550 °C thin film exhibited the peak of C−C bonding in at 283.9 eV, which was equivalent to the planar‐triangle sp^2^ bonding.^[^ ^38^ ^]^ The peak at 287 eV indicated that the sp^2^ C atom was coordinated with three N atoms,^[^ ^13^ ^]^ forming the main bonding in polyheptazine graphitic structure. The lower number of N atoms coordination presented in 600, 500, and 450 °C thin films contributed to the peak at 285.4 eV, which corresponded to the (C−N) bonding.^[^ ^39^ ^]^ Especially, the 500 °C thin film contained a significant amount of (C≡N) bonding, which was assigned to the peak at 286.4 eV and consistent with its 1D texture (Figure 2c,d). These findings can be elucidated by the thermal effects on the equilibrium between decomposition and polycondensation. The polycondensation of 2D‐layered structure was enhanced at temperature of 550 °C, while higher or lower temperatures led to dominant decomposition into 3D tetrahedral carbon.

In particular, the superhydrophilic thin films deposited at 600 and 500 °C contained C−O bonds, indicated by the peak at 281 eV, and the peak at 395 eV was assigned to the N−H bond energy in (NH_4_^+^) groups. In contrast, 550 and 450 °C thin films presented only bonds between C atoms in CN hexagonal rings with amine groups (NH_*x*_, *x* = 1, 2), as well as bonds between N and H in NH_*x*_, corresponding to the peaks at 288.5 and 400 eV, respectively. This observation further supported the conclusion that O‐based and NH_*x*_ functional groups coexisted in the CN_*x*_ thin films and that the superhydrophilicity was mainly contributed by the rich O‐based functional groups embedded on thin films deposited at 500 and 600 °C.

For comparison, the XPS results of g‐C_3_N_4_ powder samples were provided in Figure S7, Supporting Information. Unlike thin films, the powder products were located at the higher temperature position in the CVD synthesis process (See schematic in Figure 1a). The higher thermal energy in polycondensation of powder promoted a stronger polymeric binding, as evidenced by the dominance of σ bonds between C atom with one (285.8 eV) or three (286.9 eV) N atoms in the graphitic structure. In contrast, at the lower annealed temperature like 450 °C, the decomposition reaction of precursor was slower and the polymerization of abundant melamine in precursor was not totally completed, as evidenced by the lighter yellow color of powder sample (Figure S2a, Supporting Information). The conjugated bonds in CN hexagonal rings were determined by the peaks at 401.7 and 402.7 eV. Moreover, the functional groups were demonstrated by the peak of C=O (290 eV), N−H in the amines (400 eV), and N−O in the nitrates (403–405 eV). All the g‐C_3_N_4_ powder samples contained large O‐based functional groups, which was directly relevant to the superhydrophilic membranes made from powder samples. The variety of functional groups in both thin films and powder samples resulted from the multielemental dry air flow during annealing process. The constant flow partially removed NH_3_ gas, which was released from decomposition of precursor, and maintained a constant ratio of N_2_, O_2_, CO_2_, and Ar during the polycondensation of CN_*x*_ thin films and g‐C_3_N_4_ powder. Therefore, the different functional groups implanted to surface can be simply controlled by temperature without affecting intrinsic optical properties of g‐C_3_N_4_ structure (Figure S8, Supporting Information) except their morphologies. The thin film synthesized at 500 and 600 °C showed their surface roughness increase (Figure S9, Supporting Information) that agreed with their chemical hydrophilicity increase (see Figure 2b).

The atomic percentage (at%) of carbon (C), nitrogen (N), and oxygen (O) in CN_*x*_ thin films were strongly influenced by annealing temperature (Table S2, Supporting Information). Overall, the N at% increased proportionally with annealing temperature, while the O at% decreased, and the C at% approximately maintained at 42%, except for the 450 °C thin film. The low N at% of 450 °C thin film was due to the low thermal energy, where the decomposition reaction was stronger than polymerization, as in above‐mentioned TGA result. The superhydrophilic 500 °C thin film had high O concentration contributed by rich O‐based functional groups (=O, –OH and –NO_*x*_). Meanwhile, the C/N ratio in 550 and 600 °C thin films was similar to the g‐C_3_N_4_ powder. In contrast, annealing temperature was less effective in controlling the at% in g‐C_3_N_4_ powder (Table S3, Supporting Information). The graphitic powder has C/N ratio of 1.05–1.15 with small amount of oxygen intercalation (4.84–7.39 at%).

The wettability of CN_*x*_ thin films was highly consistent with their chemical bonding. The superhydrophilicity in 600 and 500 °C thin films was associated with the rich O‐based functional groups (indicated by C−O and C=O bonds) on the surface, resulting in high attraction to water molecules. Meanwhile, 550 °C is the ideal temperature for the polycondensation of heptazine structure and 450 °C is for the combined structure of triazine and heptazine (see Figure 3a,b). The well‐established structure of 450 and 550 °C samples had fewer active sites to attach O‐based functional groups except the NH_*x*_ groups which persisted in melamine. In contrast, the sample synthesized at 500 °C was in the intermediate state of transforming triazine to heptazine, and sample synthesized at 600 °C started decomposing heptazine to tetrahedral carbon; therefore, they had high level of defect/active sites for O‐based functional groups embedding. Moreover, the 1D texture on 2D surface of 500 °C thin film was relevant to the abundance of C≡N bonds which contributed to the 1D structure alignment (Figure 2c,d) which significantly increase its chemical wettability as well. Further, the superhydrophilicity of the g‐C_3_N_4_ membranes was subjected to C=O groups in powder samples, according to FT‐IR and XPS results (Figure 3c and 4). Apart from that, CA of 450, 500, and 600 °C g‐C_3_N_4_ membranes with water degraded to nearly 0° within 2 s; meanwhile, the CA of 550 °C membrane made a quick drop from 30° to 6°. The differences were related to the appearance of NO_*x*_ and OH functional groups in the powder formed at 450, 500, and 600 °C, instead, the NH_*x*_ groups in 550 °C membrane (Figure S7, Supporting Information) had lower polarization toward the hydrogen bonding with water molecules than the NO_*x*_ and OH groups.

**Figure 5:**
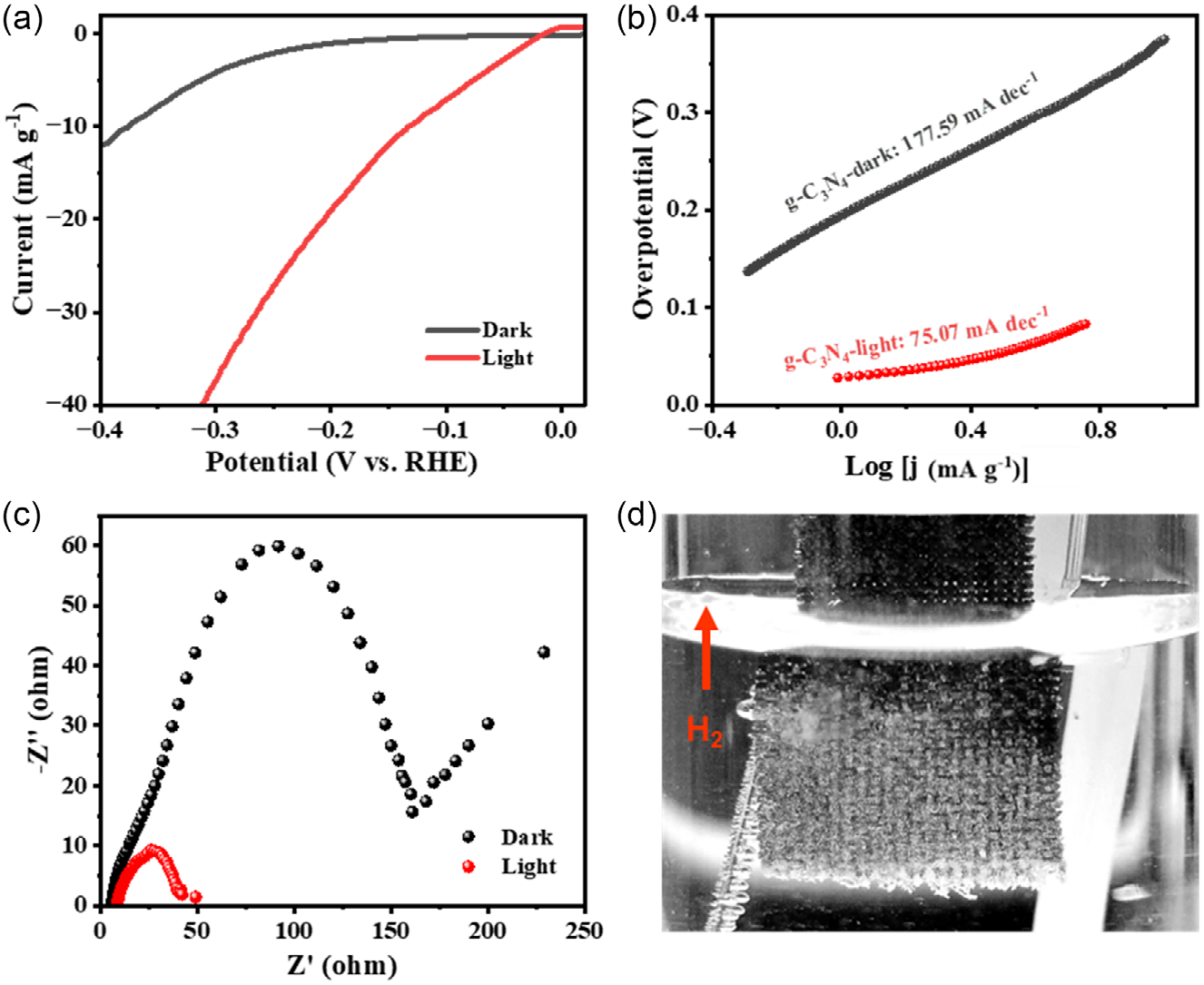
Photoelectrocatalytic performance of superhydrophilic carbon nitride. a) Polarization curves, the corresponding, b) Tafel slope, and c) Nyquist plots of electrochemical impedance spectroscopy of prepared CN_*x*_@CC electrode under dark and light (15 W) condition. d) Digital picture of generating H_2_ bubbles on electrode.

Superhydrophilicity can benefit the photoelectrocatalytic performance by increasing ion transfer and gas separation.^[^ 19, 20 ^]^ In addition, the superhydrophilicity can be further enhanced under light irradiation (Figure S10, Supporting Information). The HER performance of superhydrophilic CN_*x*_ thin film directly deposited on carbon cloth (CN_*x*_@CC) was examined through linear sweep voltammetry (**Figure** **5**a) under 15 W Xenon arc lamb irradiation (see Experimental Section). The CN_*x*_@CC showed an overpotential of 373.7 mV and a Tafel slope of 177.59 mA dec^−1^ under dark condition (Figure 5b). Noted that CN_*x*_ is an important photocatalysts with a narrow bandgap, its activity great improved under light irradiation with a reduced overpotential of 130.1 mV and a lower Tafel slope of 75.07 mA dec^−1^, surpassing that the values in previous work.^[^ 1, 40, 41, 42 ^]^ The electrochemical impedance spectroscopy (Figure 5c) revealed the electron transfer kinetics of the electrode, low ohmic resistances were realized under both dark and light conditions, which was ascribed to the superhydrophilic of the synthesized CN_*x*_. Besides, the superhydrophilic surface reduces the bubbles size and promotes the detachment of bubbles from the electrode.^[^ ^43^ ^]^ Figure 5d and the Movie S1, Supporting Information, recorded that H_2_ bubbles generated on the electrode are small and can be released quickly, which effectively avoided dead surface area and increased current density. To investigate the relationship between hydrophilicity and photoelectrochemical performance, the electrocatalytic performance of CN_*x*_@CC grown under different synthesis temperature is tested and provided in Figure S11, Supporting Information. The 450 °C CN_*x*_@CC shows a lowest overpotential of 277.0 mV under light condition, correspondingly, it has the worst hydrophilicity, as depicted in Figure 2. With the increase of hydrophilicity, the photocatalytic performance was improved. It is evidenced that the superhydrophilicity of catalyst is feasible for the improvement of the HER performance.

## 3 Conclusions

We have successfully synthesized the superhydrophilic CN_*x*_ thin films and g‐C_3_N_4_ membranes using direct CVD method. The superhydrophilicity of our products breaks the previous limitation of the as‐grown CN_*x*_ products.^[^ 24, 26 ^]^ The effect of different temperature conditions on the thermal polycondensation of CN_*x*_ in dry air medium has been revealed, suggesting the dominant incorporation of O‐based or N‐based functional groups can be spontaneously achieved by CVD annealing, followed by the relative change in chemical wettability. Moreover, enriched functional groups as well as (C≡N) bonds collectively promoted the formation of 1D texture that greatly enhanced the surface area and porosity of CN_*x*_ thin films. The superhydrophilic CN_*x*_ thin films and the g‐C_3_N_4_ powder are ideal candidates for a variety of applications working in water/humid environments.

## 4 Experimental Section

4.1

4.1.1

##### Sample Preparation: Synthesis of Carbon Nitrides

About 0.6 g of guanidine carbonate (linear formula NH_2_C(=NH)NH_2_·½H_2_CO_3_, 99%, Merck 593‐85‐1) was placed at the end of a quartz test tube (length: 155 mm, diameter: 12 mm). A glass microscope slide or carbon cloth (CC) was positioned 1 cm away from the guanidine carbonate. The test tube was sealed with quartz wool and put into a tube furnace nameplated STF 15/180. The furnace was heated up at the speed of 10 °C min^−1^ from room temperature to the desired temperature and annealed for 2 h afterward. The dry air flow (78.09% nitrogen, 20.95% oxygen, 0.93% argon, and 0.04% carbon dioxide) of 100 mL min^−1^ was supplied constantly from 30 min before the heating process until the end of synthesis process.

##### Sample Preparation: Preparation of Graphitic Carbon Nitride (g‐C_3_N_4_) Membrane

About 10 mg of g‐C_3_N_4_ powder dispersed in 10 mL of dimethylformamide (DMF) solvent (99.8%, Merck 68‐12‐2) was placed into ultrasonic bath for an hour (37 kHz, 80% power) before being filtrated on hydrophilic polytetrafluoroethylene (PTFE) membrane with pore size of 0.1 μm to form g‐C_3_N_4_ membranes (see Figure S2, Supporting Information).

##### Characterization Techniques: Thermogravimetric Analysis

The TGA measurement was carried out using the thermogravimetric analyser (TA Instruments Q500). About 15 mg of guanidine carbonate salt was put to an alumina pan hanging in the TGA chamber. A dry air flow of 20 mL min^−1^ was supplied to the TGA chamber in whole measuring process to imitate the synthesis conditions. At first, the chamber was equilibrated at 100 °C for 30 min to remove humid absorption. Then the chamber was ramping up of 10 °C min^−1^ to 600 °C and isothermal for 30 min before naturally cooling down to room temperature.

##### Characterization Techniques: Contact Angle Measurements

The wetting behaviors of the samples were examined by CA measurement, using sessile drop technique (Drop shape analyser DSA25S, KRÜSS GmbH, Germany). It was performed under ambient conditions (20 °C in temperature, 50% in humidity). A water droplet of 1–5 μL was deposited on a substrate and CA was measured within two seconds. The CA analysis from the recorded videos was processed by the software of ImageJ.

##### Characterization Techniques. X‐ray Diffraction

XRD results of thin films and powder were carried out by the Smartlab X‐Ray diffractometer (RIGAKU, Japan), which scanned over the sample in the 2*θ* range of 10°–60°, with the resolution of 0.02°.

##### Characterization Techniques: Fourier Transform Infrared Spectroscopy

FTIR spectra of sample were recorded using FT/IR‐4700 (JASCO, Japan) spectrophotometer, which scanned over the wavenumber range of 600–4000 cm^−1^, with resolution of 1 cm^−1^ in the transmittance mode.

##### Characterization Techniques: X‐ray Photoelectron Spectroscopy

XPS was applied to measure the chemical binding energy as well as the atomic ratio (at%) using an Al X‐ray source (Thermo‐Scientific, ESCALAB 250Xi). All the peaks were measured under high vacuum (10^−8^ Torr). The raw XPS data had been corrected using Shirley method^[^ ^44^ ^]^ to subtract signal from the inelastic scattering of electrons before analysis (See Figure S10, Supporting Information).

##### Characterization Techniques: Scanning Electron Microscopy

SEM (Philips FEG SEM XL30, USA) was used to examine the cross‐section morphology of the thin film. High‐level resolution at different magnifications was obtain by operating at different accelerating voltages.

##### Characterization Techniques: Atomic Force Microscopy

AFM measurement was carried out using AFM5300E system (Hitachi, Japan). The tapping mode was applied for observation of the topography, using a gold‐coated Si cantilever (NSG30, Nanotips, *f* = 340 KHz, C = 1 N m^−1^).

##### Characterization Techniques: Photoelectrochemical Measurement

The photoelectrochemical measurements were performed by a three‐electrode system in 0.5 m H_2_SO_4_ electrolyte. The in situ grown CN_*x*_@CC is used as the work electrode, Ag/AgCl and Pt are acted as the reference and counter electrode, respectively. The HER performance was tested by linear sweep voltammetry (LSV, 10 mV s^−1^) and electrochemical impedance spectroscopy (EIS) on CHI760E electrochemical workstation. Photocurrents were obtained using a 300 W Xenon arc lamp under an output power of 15 W. All the polarization curves were obtained without IR correction.

## Conflict of Interest

The authors declare no conflict of interest.

## Supporting information

Supplementary Material

## Data Availability

The data that support the findings of this study are available from the corresponding author upon reasonable request.
