# Supplementary material for: Superhydrophilic 2D Carbon Nitrides Prepared by Direct Chemical Vapor Deposition
Source: Small Sci. 2023 Mar 9;3(4):2200099. doi: 10.1002/smsc.202200099 (PMC11936000; doi:10.1002/smsc.202200099)
Supplement: Supplementary file 1 — Supplementary Material [file SMSC-3-2200099-s001.zip › smsc202200099-sup-0001-SuppData-S1.pdf]

Supporting Information

Superhydrophilic Two-Dimensional Carbon Nitrides Prepared by Chemical Vapor Deposition

Quoc Huy Thi[1,2], Ping Man[1,2], Lingli Huang[1,2], Xin Chen[1,2], Jiong Zhao[3,4], Thuc Hue Ly*[1,2]*

[1] Department of Chemistry and Center of Super-Diamond & Advanced Films (COSDAF), City University of Hong Kong, Kowloon, Hong Kong, China.

[2] City University of Hong Kong Shenzhen Research Institute, Shenzhen, China.

[3] Department of Applied Physics, The Hong Kong Polytechnic University, Kowloon, Hong Kong, China.

[4] The Hong Kong Polytechnic University Shenzhen Research Institute, Shenzhen, China.

This material contains Supplementary Figure S1 – S8 and Table S1 – S3.

SUPPORTING FIGURES AND TABLES

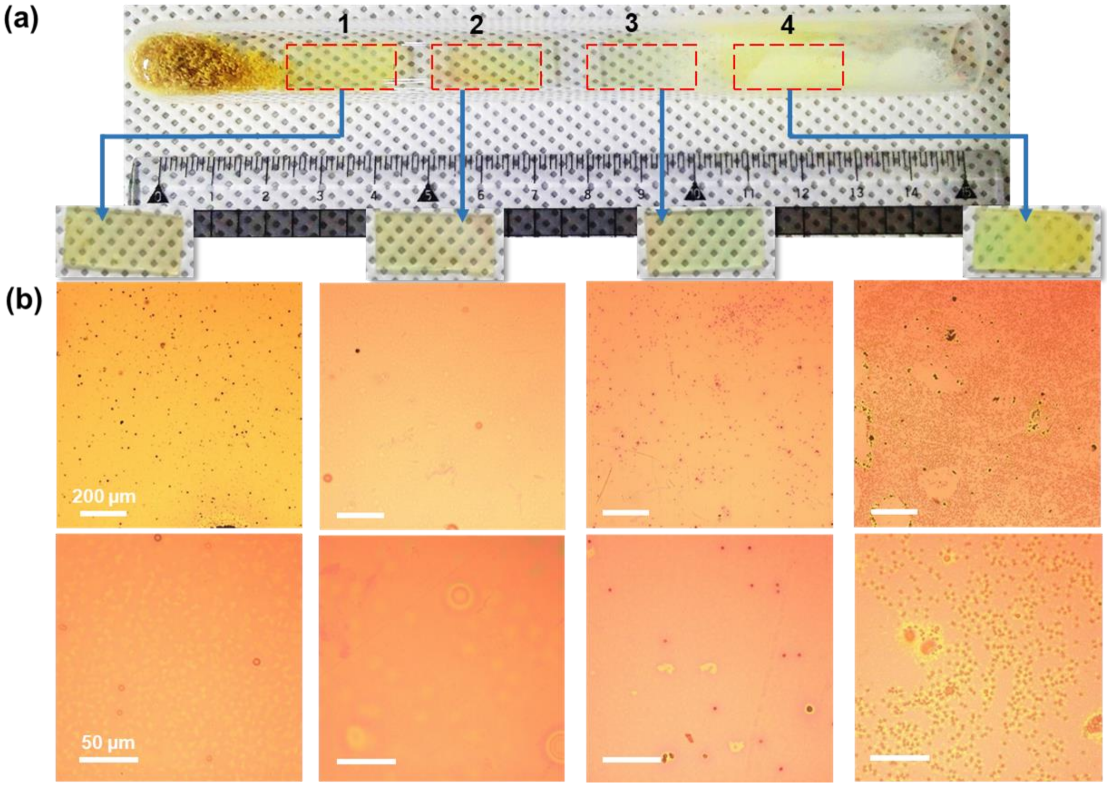

Figure S1. **Optimization of substrate position. (a)** Digital images and **(b)** optical microscope (OM) images of CN_x thin films deposited on glass substrates were dispensed at different locations in the test tube. The sample in position 2 which was 3 – 5 cm away from precursor shows the cleanest surface and uniform transparent yellow thin film on glass substrate.

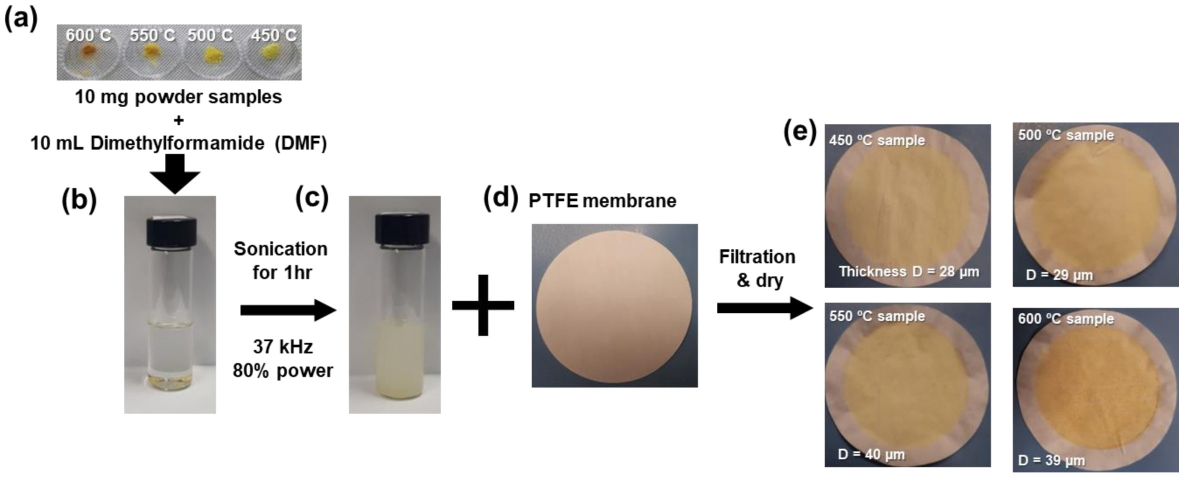

Figure S2. **Preparation of membranes from powder sample. (a)** The digital pictures of powder samples annealed at different temperature. About 10 mg of selected powder sample at first was dispersed in **(b)** 10 mL dimethylformamide (DMF). **(c)** The vial contained sample solution was put into a sonication bath at 37 kHz – 80% power in 1hrs for well dispersion. **(d)** Then the solution was filtrated through a hydrophilic PTFE filter to form a uniform membrane on the filter. **(e)** The pictures of membranes were dried thoroughly in oven at 60 ºC for further measurement.

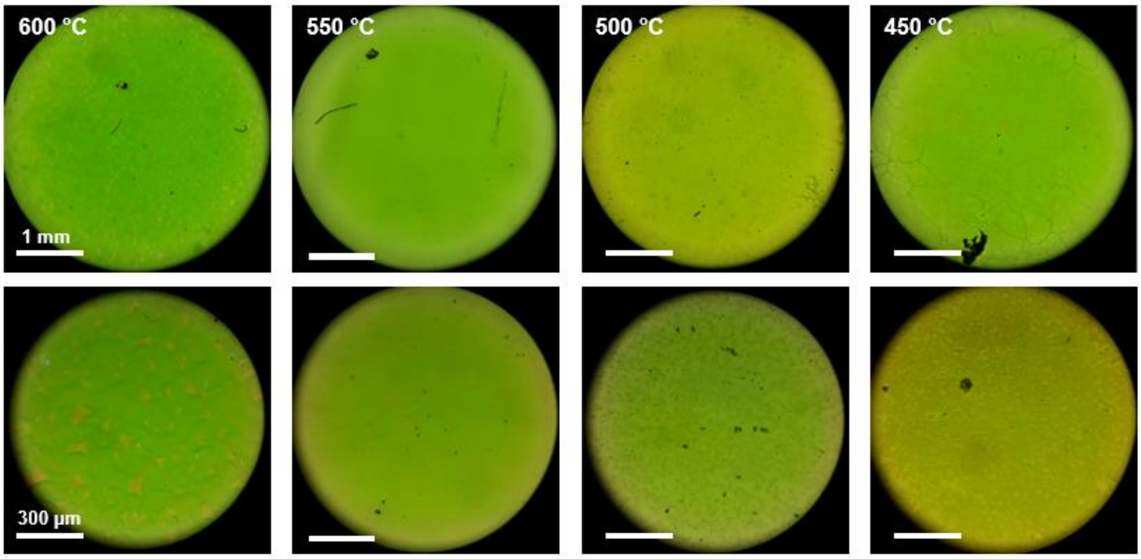

Figure S3. **The comparison of annealing temperature.** Backlight OM images of CN_x thin films deposited at different annealing temperatures.

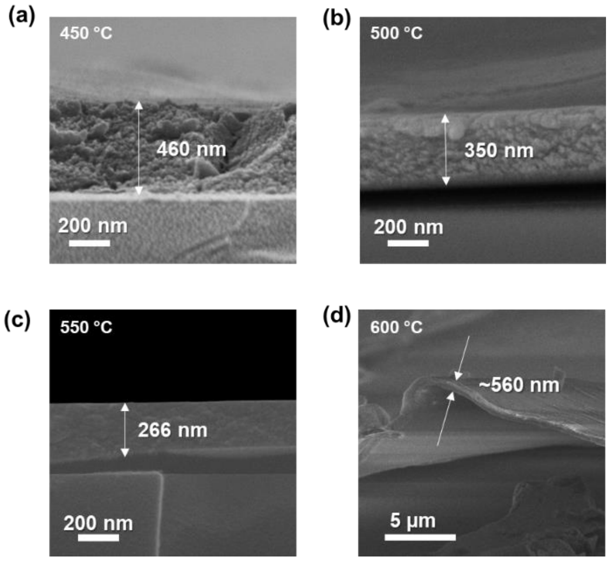

Figure S4. **Scanning electron microscope (SEM) images of the cross-sectional morphology. (a-d)** The cross-sectional SEM images of CN_x thin film deposited at 450, 500, 550 and 600 ºC, respectively.

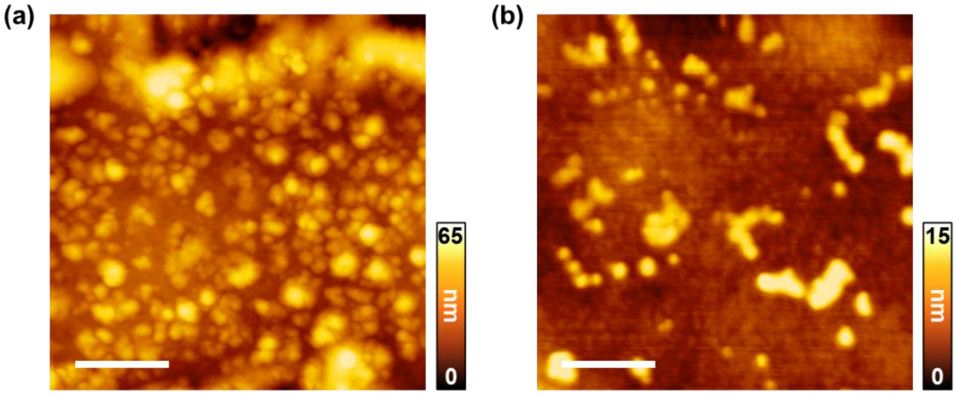

Figure S5. **Atomic microscope (AFM) topographic images of the CN_x thin films. (a, b)** The AFM topographic images of CN_x thin film deposited at 450 and 550 ºC, respectively. Scale bar 500 nm.

Table S1. Surface roughness and contact angle with water of CN_x thin films deposited at different annealing temperature

Thin films surface	450 °C	500 °C	550 °C	600 °C
AFM Roughness (RMS)	10.4	4.3	2.5	-
Contact angle (°) 0 sec	26.25	12.75	28.75	15.5
1st sec	22	8.25	19	8.5
2nd sec	21.25	6.75	15	5.5

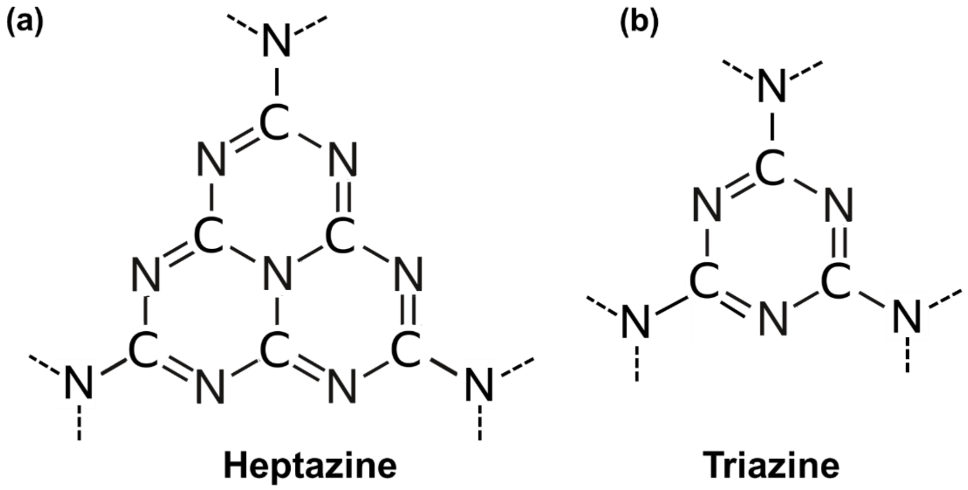

Figure S6. **The basic unit of graphitic carbon nitrides (g-C_3N_4). (a)** Heptazine and **(b)** Triazine.

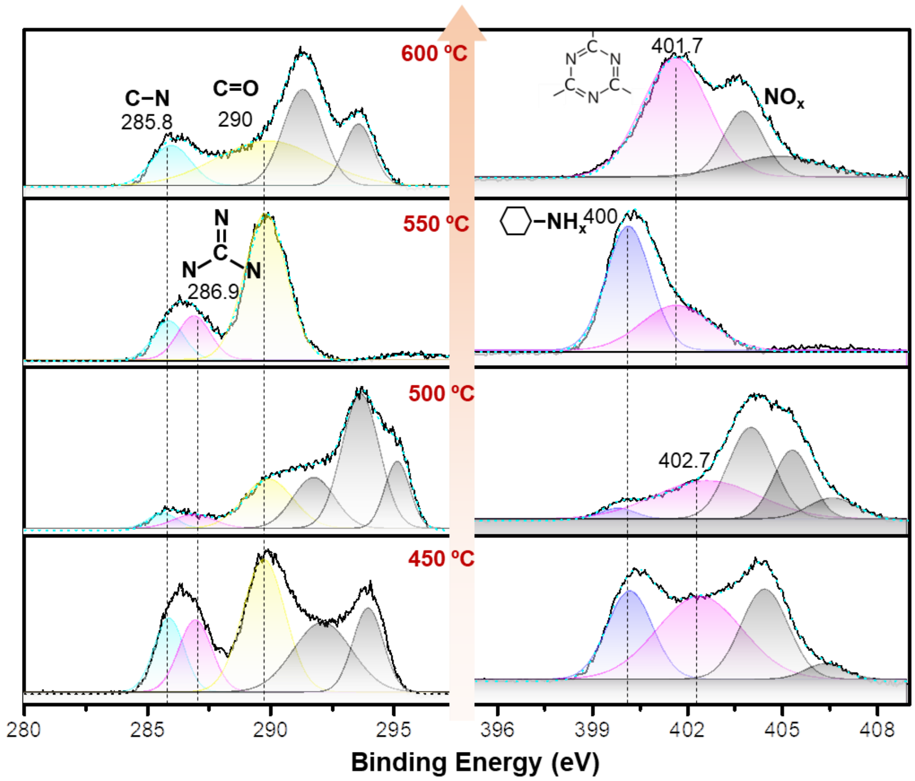

Figure S7. **X-ray photoelectron spectroscopy (XPS) characterizations of g-C_3N_4 powder sample synthesized at different annealing temperature.** The XPS spectrum peak fitting of carbon 1s core-level (left) and nitrogen 1s core-level (right) of g-C_3N_4 synthesized at 450, 500, 550 and 600 ºC.

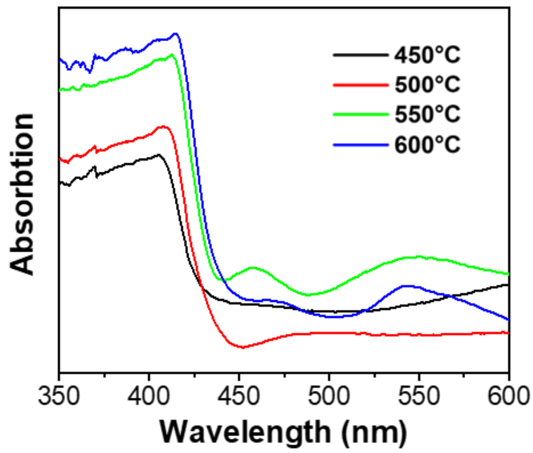

Figure S8. **Absorption spectrum of CN_x thin films deposited at different annealing temperature.** The absorption edge at 420 – 440 nm light agreed with the optical bandgap of graphitic structure.

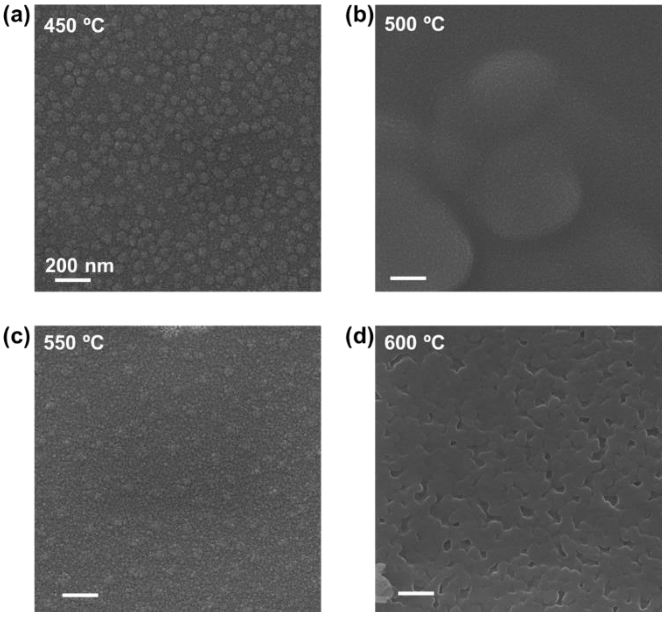

Figure S9. The top-view SEM images of CN_x thin films deposited on glass substrates at **(a)** 450 ºC, **(b)** 500 ºC, **(c)** 550 ºC and **(d)** 600 ºC.

	Light off	Light on
CNx-550 ℃	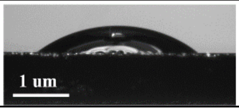	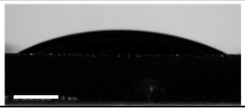
Contact angle	37.721	26.859

Figure S10. Hydrophilicity of the sample (550 ℃ CN_x) on glassy carbon electrode without and under light. Scale bar = 2 mm. The CA is collected when the droplets make first contact.

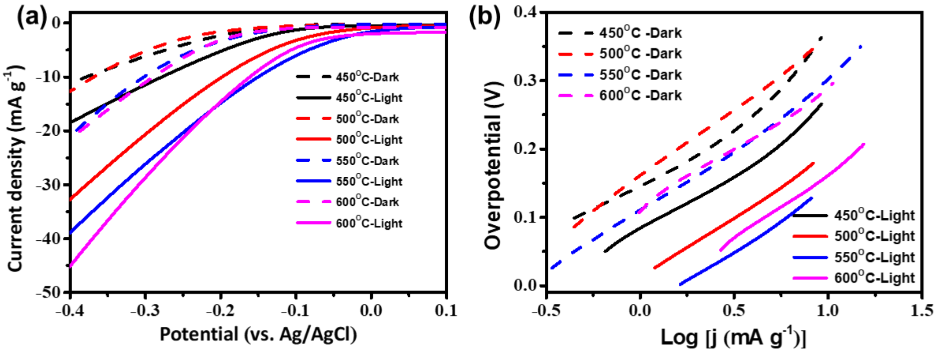

Figure S11. Photoelectrocatalytic performance of CN_x sample synthesized at different temperature (450, 500, 550 and 600℃). (a) Polarization curves and the corresponding (b) Tafel slope of prepared CN_x @CC electrodes under dark and light (13 W) condition.

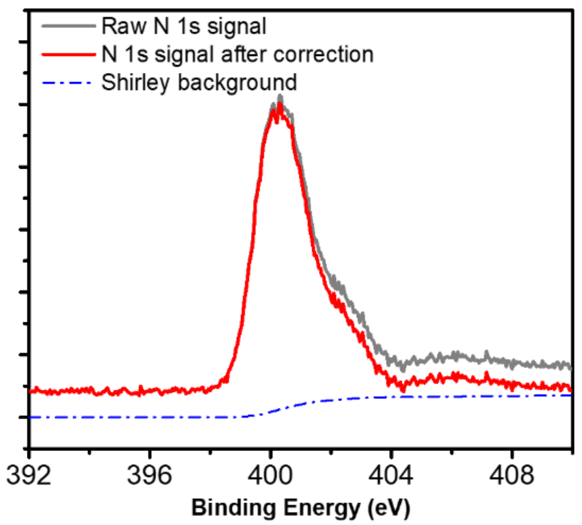

Figure S12. The XPS signal of N 1s of g-C_3N_4 powder synthesized at 550 ºC before and after subtracting the signal from the inelastic scattering of electrons using Shirley method.

Table S2. Elemental atomic ratio of CN_x thin films deposited at different annealing temperatures.

Element	Synthesis temperature			
	**600˚C**	**550˚C**	**500˚C**	**450˚C**
C (at%)	41.17	42.86	42.42	58.06
N (at%)	47.94	41.23	30.02	14.92
O (at%)	10.44	15.25	27.26	26.7
C/N ratio	0.86	1.04	1.41	3.89

Movie S1: A video of showing H_2 bubbles generated on the suphydrophilic CN_x@CC electrode are small and can be released quickly, which effectively avoided dead surface area and increased current density.

Table S3. Elemental atomic ratio of g-C_3N_4 powder synthesized at different annealing temperatures.

Element	Synthesis temperature			
	**600˚C**	**550˚C**	**500˚C**	**450˚C**
C (at%)	48.69	49.30	48.65	50.9
N (at%)	45.17	43.30	46.22	44.26
O (at%)	6.14	7.39	5.14	4.84
C/N ratio	1.08	1.14	1.05	1.15
